# Ehlers-Danlos Syndrome, Hypermobility Type: Impact of Somatosensory Orthoses on Postural Control (A Pilot Study)

**DOI:** 10.3389/fnhum.2017.00283

**Published:** 2017-06-08

**Authors:** Emma G. Dupuy, Pascale Leconte, Elodie Vlamynck, Audrey Sultan, Christophe Chesneau, Pierre Denise, Stéphane Besnard, Boris Bienvenu, Leslie M. Decker

**Affiliations:** ^1^COMETE, INSERM, UNICAEN, Normandie UniversitéCaen, France; ^2^Department of Internal Medicine, University Hospital Center of Caen, UNICAEN, Normandie UniversitéCaen, France; ^3^LMNO, CNRS, UNICAEN, Normandie UniversitéCaen, France

**Keywords:** subjective vertical, proprioception, compressive garments, proprioceptive insoles, postural sway

## Abstract

Elhers-Danlos syndrome (EDS) is the clinical manifestation of connective tissue disorders, and comprises several clinical forms with no specific symptoms and selective medical examinations which result in a delay in diagnosis of about 10 years. The EDS hypermobility type (hEDS) is characterized by generalized joint hypermobility, variable skin hyperextensibility and impaired proprioception. Since somatosensory processing and multisensory integration are crucial for both perception and action, we put forth the hypothesis that somatosensory deficits in hEDS patients may lead, among other clinical symptoms, to misperception of verticality and postural instability. Therefore, the purpose of this study was twofold: (i) to assess the impact of somatosensory deficit on subjective visual vertical (SVV) and postural stability; and (ii) to quantify the effect of wearing somatosensory orthoses (i.e., compressive garments and insoles) on postural stability. Six hEDS patients and six age- and gender-matched controls underwent a SVV (sitting, standing, lying on the right side) evaluation and a postural control evaluation on a force platform (Synapsys), with or without visual information (eyes open (EO)/eyes closed (EC)). These two latter conditions performed either without orthoses, or with compression garments (CG), or insoles, or both. Results showed that patients did not exhibit a substantial perceived tilt of the visual vertical in the direction of the body tilt (Aubert effect) as did the control subjects. Interestingly, such differential effects were only apparent when the rod was initially positioned to the left of the vertical axis (opposite the longitudinal body axis). In addition, patients showed greater postural instability (sway area) than the controls. The removal of vision exacerbated this instability, especially in the mediolateral (ML) direction. The wearing of orthoses improved postural stability, especially in the eyes-closed condition, with a particularly marked effect in the anteroposterior (AP) direction. Hence, this study suggests that hEDS is associated with changes in the relative contributions of somatosensory and vestibular inputs to verticality perception. Moreover, postural control impairment was offset, at least partially, by wearing somatosensory orthoses.

## Introduction

The Ehlers-Danlos syndrome (EDS) is a heterogeneous group of hereditary connective tissue diseases, which are present in at least 1/5000 individuals with a majority of women (Sobey, 2014). Degradation of the composition and elasticity of connective tissue results in a broad, pronounced and unspecific symptomatology. Consequently, the revised Brighton criteria classified EDS in six subtypes, according to the predominance of their clinical manifestations (Beighton et al., 1998). The EDS hypermobility subtype (hEDS) is the most frequently encountered. Besides common symptoms with other subtypes such as fatigue and pain, hEDS is characterized by generalized joint hypermobility combined with variable cutaneous hyperelasticity and proprioceptive impairment (Beighton et al., 1998; Castori, 2012). Indeed, few studies that have investigated proprioceptive sensitivity (i.e., joint position sense) in hEDS, have demonstrated the existence of proprioceptive impairment in this population (Rombaut et al., 2010a; Clayton et al., 2015). A strong hypothesis to explain the neurophysiological basis of this impairment suggests that the generalized joint hypermobility specific to hEDS induces excessive and repeated extension of the ligaments, which damages the surrounding proprioceptive receptors (Ruffini’s and Pacini’s corpuscles; Golgi tendon organs). Additionally, changes in cutaneous elasticity probably affects pressure information transmitted by cutaneous tactile mechanoreceptors to cortical areas. Hence, it is likely that hEDS induces not only a proprioceptive deficit but, more broadly, a somatosensory deficit. Consequently, the major functional disabilities expressed by these patients, including clumsiness and falls, which sometimes lead to kinesiophobia, could be the result of this somatosensory impairment (Rombaut et al., 2012).

Indeed, somatosensory information, arising from muscles, skin, and joints, plays a key role in perception, balance and, more broadly in movement. Currently, there is growing evidence that balance and movement are both based on heteromodal integration of three types of sensory modality, visual, vestibular, and somatosensory, which carry redundant, specific and complementary information (Massion, 1992; Lacour et al., 1997). The integration of these sensory modalities by the central nervous system provides three spatial frames of reference—egocentric (i.e., body), geocentric (i.e., gravity) and allocentric (i.e., external cues)—which contribute to the development of internal models crucially involved in balance and movement (Gurfinkel et al., 1981; Massion, 1994; Mergner and Rosemeier, 1998). In the sensorimotor processes, internal models refer to a neural process responsible for synthesizing information from sensory modalities and combine efferent and afferent information to resolve sensory ambiguity (Merfeld et al., 1999). Furthermore, sensory processing is a flexible mechanism (Peterka, 2002). The central nervous system continually modulates weight assigned to each sensory modality to provide a dynamic internal representation, making it possible to always generate an appropriate muscle response to maintain and adapt balance to the continuously changing environment (Van der Kooij et al., 2001; Zupan et al., 2002; Peterka and Loughlin, 2004; Logan et al., 2014). Within this process, the somatosensory system specifically provides information about the position of different parts of the body with respect to one another. Moreover, it allows characterization and localization of touch and pain (Dijkerman and De Haan, 2007). Thus, the somatosensory system mainly contributes to the sensorimotor map of body space in internal models, an unconscious process also called the “body schema” (De Vignemont, 2010).

Mittelstaedt (1983) reported that information provided by proprioception contributes considerably to the maintenance of body verticality. The perception of vertical is considered be the outcome of synthesizing visual, somatosensory and vestibular information (Brandt et al., 1994; Bisdorff et al., 1996; Merfeld et al., 1999; Van Beuzekom and Van Gisbergen, 2000; Bronstein et al., 2003; Barbieri et al., 2008; Pérennou et al., 2008; Tarnutzer et al., 2009). However, it is known that the contribution of each sensory modality in verticality perception varies between subjects and, to a greater extent, in populations presenting either vestibular impairments (e.g., patients with unilateral vestibular loss; Lopez et al., 2008) or somatosensory impairments (e.g., stroke patients with a hypoesthesia pressure and paraplegic patients; Barra et al., 2010). Interestingly, the Aubert effect, consisting in tilting of the visual vertical towards the body during lateral body tilt due to the resultant of the gravitational vector (i.e., perception of the otolith organ) and the idiotropic vector (i.e., perception of the main longitudinal axis of the body), is modified in favor of gravitational vector proportionally to the degree of somatosensory impairment (Barra et al., 2010). Hence, it seems reasonable to inquire whether somatosensory impairment in hEDS patients might modify the Aubert effect. At the same time, it has been previously shown that hEDS patients develop body schema disorders resulting in partial loss of movement control (Rombaut et al., 2010b) and postural instability (Galli et al., 2011). This deterioration in postural stability is manifested in both static (standing) and dynamic (walking) conditions (Rombaut et al., 2011; Rigoldi et al., 2013). Previous studies have already shown a strong connection between somatosensory impairments and balance disorders, especially in Parkinson’s disease (Jacobs and Horak, 2006; Vaugoyeau et al., 2011). Typically, these patients, as in normal aging, compensate for their sensory deficit by an overreliance on visual information (Lord and Webster, 1990; Isableu et al., 1997; Azulay et al., 2002). Therefore, one can speculate that somatosensory impairment could be responsible to a large extent for this postural instability, and that it could be compensated for by using a high level of visual information.

Compression garments (CG) have been tested empirically in clinical practice in hEDS, resulting in beneficial effects on pain, fatigue and mobility. Speculatively, the CG, due to their mechanical effect, are thought to enhance joint coaptation and increase the pressure of the subcutaneous connective tissue to a normal range. Hence, CG may enhance somatosensory feedback to the brain and, thus, its contribution to postural control. Similarly, proprioceptive insoles (PI) may enhance plantar cutaneous afferents and postural stability. Therefore, somatosensory orthoses (i.e., CG and PI) offer a therapeutic solution to reduce somatosensory impairments, however weakly evaluated. Along with these observations, previous studies have demonstrated that CG induced an improvement in knee proprioception, and PI decreased the attentional demand for gait (Clark et al., 2014; Ghai et al., 2016). Conversely, these two ortheses showed no impact in healthy young subjects, and CG appeared to induce a deterioration of postural stability in elderly subjects (Hijmans et al., 2009; Dankerl et al., 2016). In the light of these conflicting observations, we aimed to quantify the impact of these somatosensory orthoses on postural stability in a population with a specific impairment of the somatosensory system. Indeed, it seems plausible that, although the wearing of CG has probably no immediate impact on the damaged joint proprioceptive receptors, its compressive effect applied to subcutaneous connective tissue could allow better somatosensory transmission from cutaneous tactile mechanoreceptors. Hence, somatosensory deficit could be partially reduced by CG, which would compensate for joint proprioception impairment. Similarly, enhanced plantar cutaneous afferents induced by PI could increase the available sensory information for postural control.

The goal of the present study was to assess: (i) the impact of somatosensory deficit on subjective visual vertical (SVV) and postural stability; and (ii) the effects of somatosensory orthoses (i.e., CG and PI) on static postural control. We hypothesized that: (i) somatosensory impairments would modify SVV, strongly impair postural stability and increase the use of visual information; and (ii) enhancing somatosensory feedback with the orthoses would restore the balance in the use of sensory modalities, thus reducing the use of visual information, and consequently enhance postural stability.

## Materials and Methods

### Study Population

Six patients with hEDS (6 females; mean age ± SD: 37 ± 10.41 years) and six healthy, age- and gender-matched control subjects (6 females; mean age ± SD: 36 ± 11.52 years) participated in this study. Patient selection was carried out in the Internal Medicine Department of Caen University Hospital. Inclusion criteria were based on the revised Villefranche criteria, including the presence of generalized joint hypermobility, skin hyperelasticity, chronic musculoskeletal pain, and/or a positive family history (Beighton et al., 1998). Additionally, patients must have reported hypersensoriality (e.g., a low hearing threshold). Exclusion criteria were: (i) wearing of somatosensory orthoses (i.e., PI and CG); (ii) inability to maintain a minimum of postural stability in static conditions (i.e., holding an upright stance during 1 min); (iii) treatment by a physical therapist; and (iv) other pathologies that directly impact postural control (e.g., Ménière’s disease). Finally, patients were checked for vestibular disorders by ENT examination with otolithic myogenic evoked potentials, and videonystagmography. Healthy controls subjects were recruited by local phone call. Control subjects were excluded if they had a neurologic (with a special focus on vestibular disease using the Fukuda test; Fukuda, 1959) or orthopedic disorder (analysis of foot plantar pressure distributions using a podoscope) that could affect their postural stability, and a generalized disease affecting joints, or a Beighton score >4/9.

All subjects were treated in strict compliance with the Declaration of Helsinki. The protocol was approved by the CERSTAPS (Ethical Committee of Sport and Physical Activities Research), Notice Number: 2016-26-04-13, approved by the National Academic Commission (CNU) on April 26, 2016. Written informed consent was obtained from all participants.

### Instrumentation

#### Somatosensory Orthoses

**Figure 1:**
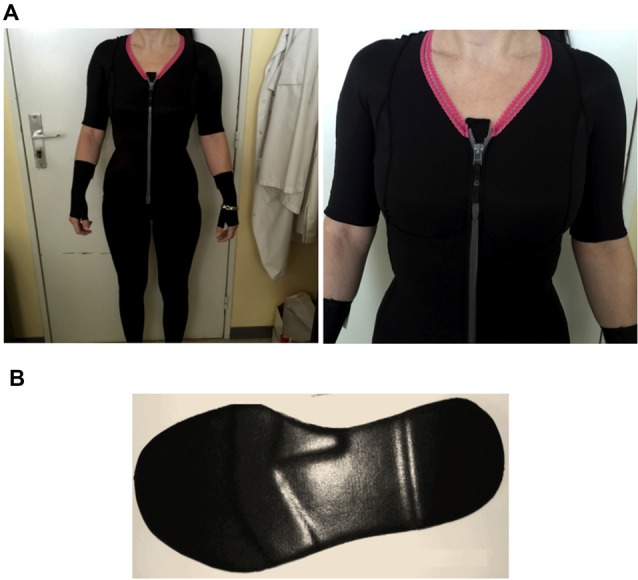
**(A)** Compression garments (CG) and **(B)** proprioceptive insoles (PI) worn by an Ehlers-Danlos syndrome hypermobility type (hEDS) patient during the experiment.

The CG and PI required in this study were customized based on the needs of each patient by orthotic and prosthetic practitioners (Novatex Medical). CG included pants, vest, and mittens, which covered the entire body of all participants (i.e., trunk, upper and lower limbs; Figure 1).

#### Postural Control

Postural sway was recorded using a motorized force platform (SYNAPSYS, France). Three strain gauges integrated into the force platform recorded the vertical ground reaction force component. The data were sampled at 100 Hz and transformed by computer-automated stability analysis software (i.e., Synapsys software) to obtain x-y coordinates of the center of pressure (COP).

#### Subjective Visual Vertical

Perception of the vertical was assessed by the SVV test using the Perspective System^®^ (Framiral^®^, France).

### Experimental Procedure

In the first part of the experiment, participants underwent postural control assessment (duration: 1 h 45 min for patients, and 20 min for controls) followed by SVV assessment (duration: 15 min for all participants).

#### Subjective Visual Vertical Assessment

To assess the SVV, each participant, in a completely darkened room, was shown, in front of them, the projection of a luminous rod (laser line 2 m in length placed 3 m in front of them). Participants could rotate the rod around its center in the clockwise or counterclockwise directions using a transmitter, and were instructed to place the rod vertically with respect to the true gravitational vertical. All subjects performed the SVV test in three conditions: standing, sitting and lying on their right side. In this latter condition, participants lay in a standard position on a stretcher with an adjustable head-rest, which was positioned identically initially for each participant (body and head were tilted, respectively, at 90° and 72°). Subjects were asked to minimize their movements during the tests. Each condition comprised four trials: two with the rod initially oriented to the right side (i.e., 30° to the right—clockwise) and two to the left side (i.e., −30° to the left—counterclockwise). The tests and conditions were randomly distributed within each participant.

#### Postural Control Assessment

**Figure 2:**
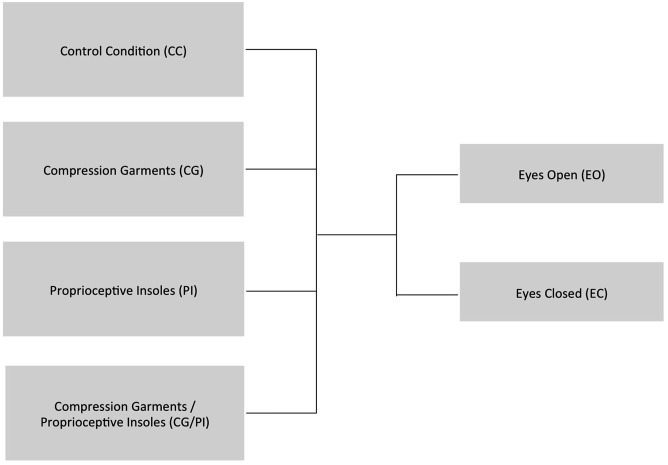
Design of the postural control assessment.

Postural sway was measured for 52 s while participants stood on a force platform. Participants were asked to stand still, barefoot, arms hanging freely, feet positioned at an angle of 30°, and to focus on a visual reference mark fixed 1.5 m in front of them in their individual line of vision. The assessment comprised four conditions with two tests each lasting 52 s, with a 20 s rest between each test, and 5 min between each condition. The start and stop signals were given 3 s before and 3 s after each acquisition. The four conditions were: (1) control condition (CC; without orthoses); (2) CG; (3) PI; and (4) the combination of CG and PI (CG-PI). Each condition was performed with either eyes open (EO) or eyes closed (EC). Participants also underwent dual-task (combining postural control with a cognitive task) and dynamic (sinusoidal translation of support) trials under the four above-mentioned conditions (results are not included in the present article). To minimize any order effects during testing, such as fatigue effects, all conditions and trials (EO/EC) were randomized among subjects. A training test was performed before testing (Figure 2).

### Data Analysis

#### Subjective Visual Vertical Analysis

SVV evaluation error was scored in degrees of deviation from the vertical. Mean errors were calculated across conditions, according to the initial orientation of the rod. Errors were scored negatively when the subjective vertical was oriented to the left, and positively when it was oriented to the right.

#### Postural Control Analysis

Postural sway parameters calculated from the COP recordings were as follows: the anteroposterior and mediolateral sway standard deviation (SD-AP/SD-ML; mm) and the sway area (AREA-CE; mm^2^) corresponding to the 95% confidence elliptic area included within the COP path.

### Statistical Analysis

The SVV (angle of deviation from the vertical) and postural (AREA-CE, SD-AP and SD-ML) dependent variables failed to display an acceptable normal distribution (Shapiro-Wilk test). Consequently, non-parametric tests were used for statistical analysis.

The Mann-Whitney U-test was used to compare healthy controls to hEDS patients on verticality perception and postural stability. A Friedman test was used to determine differences between the performances carried out in each postural condition (CC, CG, PI and CG/PI) and each SVV condition (standing, seated, lying: right and left initial orientation). When the result of the Friedman test was significant, we subsequently used a Wilcoxon test for matched samples to determine the effects of vision (EO and EC) and somatosensory orthoses on postural stability. We used the Bonferroni method to correct for multiple comparisons. Statistical significance was set at 0.05. Statistica (version 10, Statsoft, Inc., Tulsa, OK, USA) was used to perform all analyses.

## Results

### Subjective Visual Vertical

**Figure 3:**
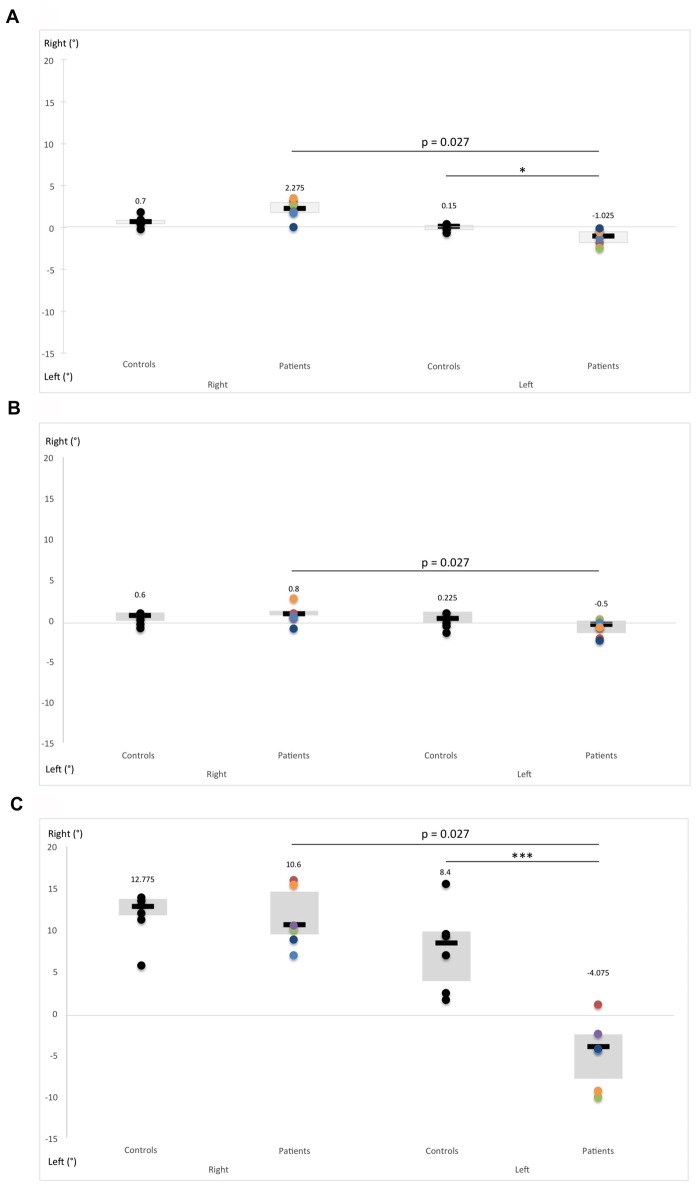
Comparison of subjective visual vertical (SVV) performance between hEDS patients and controls in different body positions: **(A)** standing, **(B)** sitting and **(C)** lying on the right side. SVV was measured by presenting a laser rod 12 times in total darkness with a 30-degree deviation from the vertical alternately on the right and the left. Subjects were asked to reposition the rod vertically using a remote control. Box plots represent median and quartiles, and dots represent performance of each participant as follows: controls: black; patient 1: red; patient 2: green; patient 3: purple; patient 4: light blue; patient 5: orange; patient 6: dark blue. *p < 0.05, ***p < 0.005.

We first analyzed perception of the visual vertical in each position (standing, seated, lying on the right side) using the Mann-Whitney U-test. In standing condition, hEDS patients oriented the vertical more in left side than controls, when the initial orientation of the rod was also on the left (*U* = 4, *p* = 0.026). Simultaneously, in lying on the right condition, when the initial orientation of the rod was on the left, patients did not exhibit the substantial perceived tilt of the visual vertical in the direction of the body tilt (Aubert effect), and oriented their vertical closer to the real vertical compared to controls, (*U* = 0, *p* = 0.002). Interestingly, in sitting condition, perception of visual vertical was similar in both groups (Figure 3).

The Friedman test revealed significant differences in verticality perception according to the initial orientation of the rod (right and left) and body position (sitting, standing and lying on the right) in hEDS patients (*p* = 0.0001) and controls (*p* = 0.00034). As 30 side-by-side comparisons were carried out for each *post hoc* analysis, the Bonferroni method was used to correct the significance level at 0.0016. Consequently, all the results from the Wilcoxon test reported below with a *p* > 0.0016 have been used because of our small sample size, and thus have a descriptive vocation.

Regardless of the position, the initial orientation of the rod seems to influence the verticality perception of hEDS patients (sitting: *Z* = 2.20, *p* = 0.027; standing: *Z* = 2.20, *p* = 0.027; lying: *Z* = 2.20, *p* = 0.027). When the initial orientation of the rod was to the right, patients showed a greater degree of deviation of verticality perception in standing compared to sitting (*Z* = 2.20, *p* = 0.027), and to a larger extent, when lying compared to sitting (*Z* = 2.20, *p* = 0.027) and standing (*Z* = 2.20, *p* = 0.027). In contrast, no difference was observed when the initial orientation of the rod was to the left. Likewise, in controls, the initial orientation of the rod did not influence verticality perception. In addition, controls presented a greater deviation of their verticality perception when lying as opposed to sitting and standing, regardless the initial orientation of the rod (right initial orientation: sitting vs. lying: *Z* = 2.20, *p* = 0.027, standing vs. lying: *Z* = 2.20, *p* = 0.027; left initial orientation: sitting vs. lying: *Z* = 2.20, *p* = 0.027, standing vs. lying: *Z* = 2.20, *p* = 0.027).

### Postural Control without Somatosensory Orthoses

**Figure 4:**
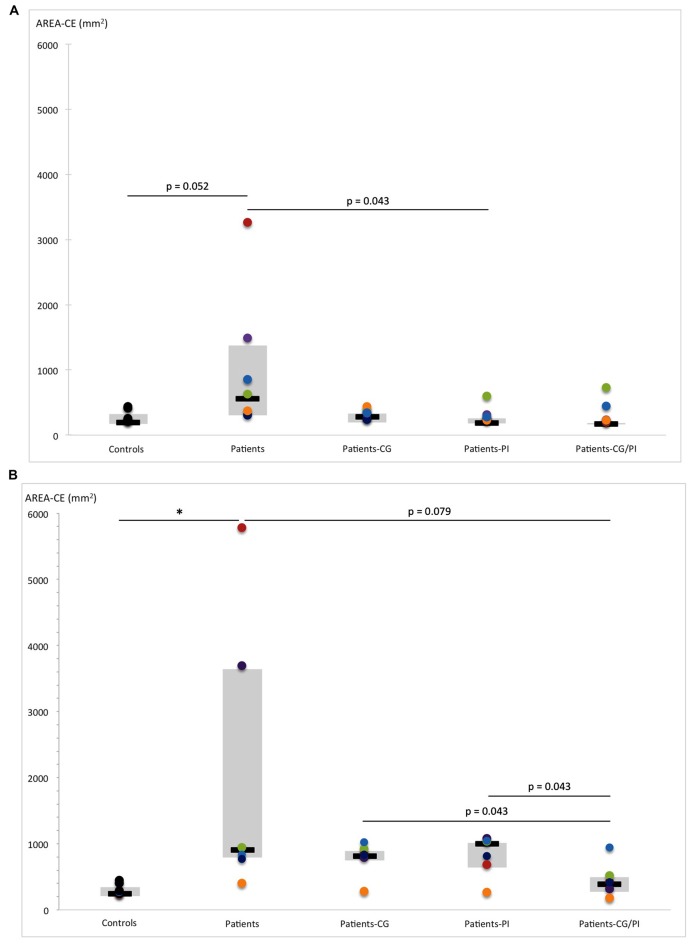
Comparison of AREA-CE (area of 95% confidence circumference, mm^2^) obtained by hEDS patients and controls, with and without somatosensory orthoses (CG, compression garments; PI, proprioceptive insoles; CG-PI, both somatosensory orthoses): in **(A)** eyes-open, and **(B)** eyes-closed conditions. Box plots represent median and quartiles, and dots represent performance of each participant as follows: controls: black; patient 1: red; patient 2: green; patient 3: purple; patient 4: light blue; patient 5: orange; patient 6: dark blue. *p < 0.05.

Compared with controls, hEDS patients showed impaired postural stability, as reflected by their increased sway area (EO, *U* = 4, *p* = 0.052) and increased AP sway SD (EO, *U* = 3, *p* = 0.015). These latter effects became more pronounced in the absence of visual information (AREA-CE: EC, *U* = 2, *p* = 0.017; SD-AP: EC, *U* = 0, *p* = 0.004). Furthermore, postural stability also deteriorated in the ML direction without vision (*U* = 4, *p* = 0.052). Besides, the Wilcoxon test comparing EO and EC revealed an increased sway area (*Z* = 2.022, *p* = 0.043) and an increased ML sway SD in hEDS patients (*Z* = 2.022, *p* = 0.043). Removal of vision had no effect on postural stability in controls (Figure 4).

### Postural Control with Somatosensory Orthoses

The Friedman test was conducted to assess the effects of somatosensory orthoses on postural stability in hEDS patients in four conditions (control, PI, CG, and PI-CG), with (EO) and without (EC) vision. Then, as six side-by-side comparisons were carried out within each *post hoc* analysis, the significance threshold was set at 0.00833, as indicated by Bonferroni correction. Similar to the SSV, all the results from the Wilcoxon test reported below with a *p* > 0.00833 have a descriptive vocation.

#### With Vision

**Figure 5:**
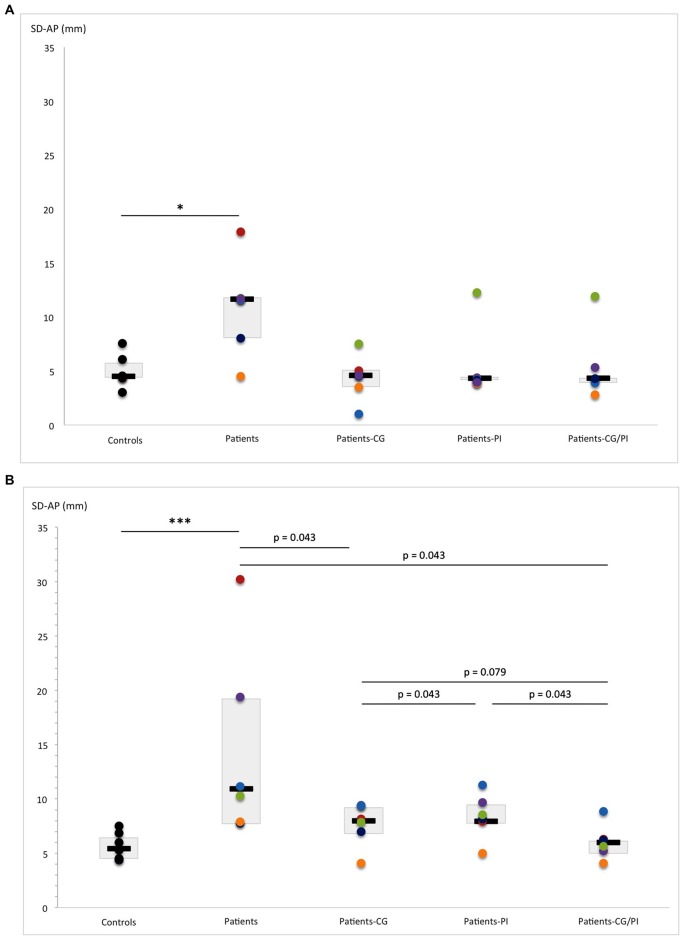
Comparison of SD-AP (standard deviation of anteroposterior center-of-pressure (COP) displacement: mm) obtained by hEDS patients and controls, with and without somatosensory orthoses (CG, compression garments; PI, proprioceptive insoles; CG-PI, both somatosensory orthoses): in **(A)** eyes-open, and **(B)** eyes-closed conditions. Box plots represent median and quartiles, and dots represent performance of each participant as follows: controls: black; patient 1: red; patient 2: green; patient 3: purple; patient 4: light blue; patient 5: orange; patient 6: dark blue. *p < 0.05, ***p < 0.005.

**Figure 6:**
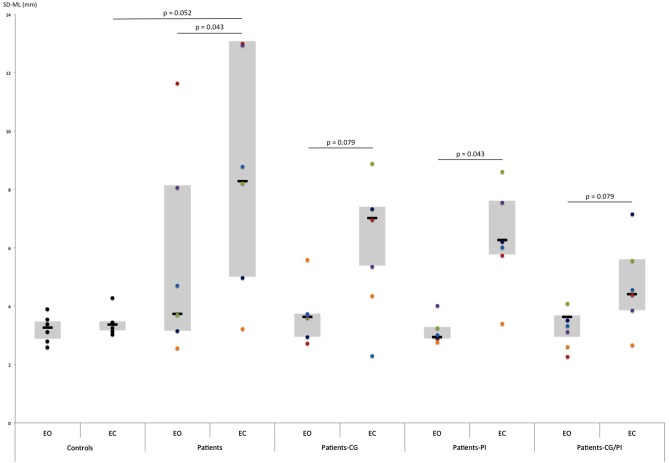
Comparison of SD-ML (standard deviation of mediolateral COP displacement: mm) obtained by hEDS patients and controls, with eyes open (EO) and eyes closed (EC) depending on the somatosensory orthoses worn (CG, compression garments; PI, proprioceptive insoles; CG-PI, both somatosensory orthoses). Box plots represent median and quartiles, and dots represent performance of each participant as follows: controls: black; patient 1: red; patient 2: green; patient 3: purple; patient 4: light blue; patient 5: orange; patient 6: dark blue.

The Friedman test revealed that somatosensory orthoses tended to have a significant effect on sway area (*p* = 0.069), with an improvement in postural stability (decreased sway area) in the presence of PI compared to the CC (*Z* = 2.022, *p* = 0.043; Figure 4A). However, the patients’ performance distribution within each orthosis condition indicates that this result may be due to a lower inter-individual heterogeneity than in CG/PI condition, and a median slightly lower than in CG condition (Figure 4A). Consequently, there is little evidence that the PI condition induced an improvement of postural stability greater than the other conditions (CG, CG/PI), which all seem to induce a beneficial but similar effect on postural stability. This effect appeared to be even more pronounced when the patient was unstable in the CC. On the other hand, somatosensory orthoses had no significant effect on AP and ML sway SD (Figures 5A, 6).

#### Without Vision

The Friedman test revealed that somatosensory orthoses significantly impacted AP sway SD (*p* = 0.040), and tended to have a significant effect on sway area (*p* = 0.06). Importantly, the simultaneous wearing of the two orthoses seems to have induced further improvement on AP sway SD (*Z* = 2.022, *p* = 0.043; Figure 5B), compared to control, as opposed to the wearing of each orthosis separately. Indeed, the observed effects were more pronounced when the two orthoses were worn together rather than separately (AREA-CE: CG vs. CG/PI: *Z* = 2.022, *p* = 0.043; PI vs. CG/PI: *Z* = 2.022, *p* = 0.043; Figure 4B; SD-AP: CG vs. CG/PI: *Z* = 1.75, *p* = 0.079; PI vs. CG/PI: *Z* = 2.022, *p* = 0.043; Figure 5B). Also, the decreased AP sway SD induced by CG (*Z* = 2.022, *p* = 0.043) tended to be greater than that induced by PI (*Z* = 1.75, *p* = 0.079; Figure 5B). However, in light of patients’ performance distribution under these two conditions, it is difficult to identify an additional effect of CG as compared to PI. In the ML direction, somatosensory orthoses did not show any significant impact on postural stability.

#### With vs. Without Vision

The increased sway area found in hEDS patients without vision and somatosensory orthoses (*Z* = 2.022, *p* = 0.043) persisted when they wore orthoses, alone (PI: *Z* = 2.022, *p* = 0.043; CG: *Z* = 1.75, *p* = 0.079) or in combination (*Z* = 2.022, *p* = 0.043). A similar result was observed for ML sway SD (PI: *Z* = 2.022, *p* = 0.043; CG: *Z* = 1.75, *p* = 0.079, PI/CG: *Z* = 1.75 *p* = 0.079; Figure 6). In contrast, visual removal did not appear to affect AP sway SD, regardless of the presence of somatosensory orthoses.

## Discussion

### Subjective Visual Vertical in hEDS

In the standing condition, the results obtained by hEDS patients suggest a greater deviation from true gravitational vertical than controls. This effect seems to be less apparent in the sitting condition. These findings suggest that hEDS is associated with changes in the neural processing of somatosensory inputs, which could in turn alter judgment of the SVV (Trousselard et al., 2003). Moreover, one can speculate that, as previously observed in stroke patients, this specific alteration of verticality perception in the standing condition could be associated with postural instability in hEDS patients, and especially with lower limb asymmetry (Bonan et al., 2006, 2007). However, correlational analyses did not strongly confirm a direct link between these two factors. The small number of subjects included in this pilot study makes these analyses irrelevant due to pronounced heterogeneity between patients in both postural stability and verticality perception performances. In addition, certain technical limitations prevented us from computing parameters able to quantify postural asymmetry. Nevertheless, these observations provide preliminary data that should be explored further. More relevantly, when lying in the right condition, the Aubert or A-effect (i.e., SVV deviation from the true vertical in the same direction as the body tilt; Aubert, 1861) was found when the rod was tilted to the right in both groups, but absent when the rod was initially left-oriented in hEDS patients. In healthy controls, the A-effect is considered to result from the subject’s tendency to shift the SVV toward the longitudinal body axis, independently of the initial orientation of the rod (Mittelstaedt and Glasauer, 1993). More specifically, it may result from changes in vestibular (i.e., otolithic organs;* gravitational vector*) and somatosensory (i.e., muscular and articulatory endocaptors, cutaneous exocaptors; interception *idiotropic vector*) inputs related to a body tilt in the dark (Bronstein, 1999). In their study, Bronstein et al. (1996) demonstrated that when patients with bilateral peripheral labyrinthine lesion are lying at approximately 90° on their right side, they presented an A-effect twice as large as controls. The authors suggested that tilt-mediated effect on the visual vertical is more likely to be of somatosensory rather than vestibular origin. The implication of the somatosensory system in verticality perception was confirmed by studies on SVV in somatosensory deficient populations (Yardley, 1990; Anastasopoulos and Bronstein, 1999). In these studies, the authors found a unilateral loss of A-effect when hemianesthetic patients were lying on the same side as their lesion, and a bilateral loss in patients with severe polyneuropathy. Thus, our results are consistent with those reported in the literature for healthy controls. A striking finding is that the perceived vertical of hEDS patients was not far from the true vertical when the rod was initially oriented to the left side. This finding is also consistent with earlier studies (Yardley, 1990; Anastasopoulos and Bronstein, 1999), and another study conducted by Barra et al. (2010), who found that the A-effect was markedly reduced in patients with somatosensory deficit (i.e., hemiplegia and paraplegia). The explanation advanced is that these patients cannot integrate somatosensory inputs. Hence, their SVV relies mainly on gravitational (vestibular) input. Another interesting finding is that this phenomenon did not appear when the rod was initially right-oriented. A plausible explanation is that, in this condition, the initial orientation of the rod was directly congruent with the joint combination of the idiotropic and gravitational vectors (internal representation of the vertical). This was not the case when the rod was initially left-oriented (i.e., rotated in a direction opposite to the longitudinal body axis). This may be due to the greater complexity of the task that led patients to preferentially rely on gravitational (vestibular) input. Indeed, to adjust the rod with their verticality perception when it was initially left-oriented, the rod systematically passed through the true vertical. Consequently, one can postulate that the predominance of vestibular input relative to somatosensory input led patients to perceive as vertical the position where the rod converged with gravitational vector. This finding is consistent with the fact that somatosensory input is not absent, but is compromised by damage to its receptors and the poor pressure transmission induced by degraded connective tissue. Hence, we could suggest that somatosensory input is also present, but its contribution to perception could be inhibited or reduced due to its lack of reliability. Finally, taken together, these findings highlight changes in the relative contributions of somatosensory and vestibular inputs to verticality perception in hEDS patients (i.e., central adaptation in somato-vestibular perceptual systems).

### Baseline Characteristics of Postural Control in hEDS

In line with previous studies, hEDS patients showed significant difficulties in controlling COP displacements (i.e., increased sway area—confidence ellipse area), especially when visual information was absent (Galli et al., 2011; Rigoldi et al., 2013). Interestingly, controls did not show any difference in their postural stability between EO and EC conditions as observed in other studies (e.g., Lacour et al., 1997; Błaszczyk et al., 2014). This result is not surprising given the fact that the healthy controls included in this study were fairly young (approximately 37 years old) and presented no orthopedic and sensory disorders. In addition, it is also possible that postural parameters used in this study were not the most sensitive to assess the effect of visual removal in healthy young subjects (Prieto et al., 1996). However, our result suggests that hEDS patients (especially the most unstable cases) relied on vision for postural stability. Marigold and Eng (2006) found that the removal of vision in stroke patients increased postural instability, particularly in the ML direction, and all the more so in presence of postural asymmetry. To explain this observation, the authors suggested that, in stroke patients, the body schema formed by the CNS may lack appropriate somatosensory information due to altered supraspinal centers (Niam et al., 1999). In hEDS patients, impairment of somatosensory receptors would induce a down-weighting of this sensory modality, compensated by an up-weighting of visual modality (slow dynamic; Chiba et al., 2015). The balance between the contributions of each sensory modality is essential in continuous sensory reweighting (fast dynamic), which permits the maintenance of efficient and adaptable postural control (Nashner, 1976; Asslander and Peterka, 2014; Chiba et al., 2015). Furthermore, hEDS patients also had great difficulty in maintaining their ML postural stability when vision was withdrawn. Therefore, ML stability appears to depend upon two factors: reliance on vision and asymmetry in postural control. Our results tend to confirm an overreliance on visual information, but suggest only the presence of postural asymmetry in hEDS patients. It would be interesting to investigate this question in future studies. Regardless of visual condition, the intergroup difference in sway SD was more pronounced in the AP direction. Increased AP sway was also found in stroke patients when somatosensory information was altered (Marigold et al., 2004). To interpret these results, the authors hypothesized that the ability to integrate information from cutaneous sensation can reduce the contribution of ankle proprioception in controlling postural sway. Consequently, the increased AP sway observed in stroke patients in whom ankle proprioception was compromised would be due to their inability to compensate by using cutaneous plantar information (Marigold et al., 2004; Marigold and Eng, 2006). However, the authors found no correlation between cutaneous plantar foot sensation and postural sway. Thus, it is still unclear how somatosensory information affects AP postural sway in stroke patients. This finding, also observed in hEDS patients with specific somatosensory impairment, suggests that AP stability results, at least in part, from accurate somatosensory information. Moreover, a previous study conducted in healthy young subjects showed that the neuromuscular system must allocate 50 percent more effort to control AP stability in the upright stance (Błaszczyk et al., 2014). Thus, the greater AP sway SD in hEDS patients suggests that they may have difficulty generating sufficient neuromuscular effort to maintain their postural stability. However, this hypothesis needs to be confirmed as Błaszczyk et al. (2014) used COP velocity (sway ratio and sway directional index) to quantify postural stability. Technological limitations prevented us from computing this parameter. Hence, we posit that the greater neuromuscular effort allocated for controlling AP stability may produce higher recruitment of the somatosensory system. Therefore, somatosensory impairment could prevent hEDS patients from producing sufficient neuromuscular effort to stabilize their balance in the AP direction. To summarize, specificities of postural control in hEDS patients appear to result from both their somatosensory impairment and the adoption of postural compensatory strategies. This imbalance in multisensory integration complicates control of the upright stance and, therefore, is at least partly responsible for their postural instability. Finally, this process seems to be relatively variable between participants. This finding was not surprising given that considerable variability in clinical expression is commonly observed in hEDS patients. Consequently, it has recently been proposed to consider hEDS as a spectrum of pathogenetically-related manifestations of joint hypermobility (Malfait et al., 2017). Hence, it would be interesting to further investigate the link between severity of clinical expression of hEDS and the evolution of the sensori-motor strategy adopted by these patients.

### Effects of Somatosensory Orthoses on Postural Control in hEDS

In hEDS patients (notably the most unstable cases), the wearing of somatosensory orthoses seems to reduce their postural instability (i.e., sway area) to such an extent that their performances became comparable to those of controls in eyes-open condition. However, further investigations will be required to confirm these preliminary observations with a larger sample. Interestingly, this effect turned out to be even more pronounced in the absence of visual information. Wearing the two orthoses in combination seems to help patients stabilize their balance and minimize their AP sway SD. Thus, the combined wearing of orthoses could induce a synergetic effect. Indeed, it seems to improve postural stability more than the wearing of the CG or PI separately in the eyes-closed condition for both sway area and AP sway SD. Therefore, one can reasonably hypothesize that the increased cutaneous plantar sensation applied by pressure on sole receptors from PI could be concurrent with the increased cutaneous sensation and joint position sense promoted by CG. Hence, the combination of CG and PI could possibly enhance the available somatosensory information and, consequently, balance, even without vision. In addition, it is noteworthy that removal of visual information increases the impact of somatosensory orthoses on postural stability, especially in the AP direction. We thus suggest that, in the EO condition, visual information compensates for the lack of somatosensory information. Consequently, the removal of vision obliges patients to rebalance their use of sensory modalities in favor of somatosensory information, thus reinforcing the somatosensory input provided by orthoses. In contrast, ML stability appears to be scarcely affected by the somatosensory orthoses and remained sensitive to visual input. This result supports our previous hypothesis, which assumed that visual information was, at least in part, responsible for ML stability. Besides, our results showed no effect of somatosensory orthoses on overreliance on visual input in hEDS patients. Also, their postural strategy, which consists in compensating their lack of somatosensory information by ample use of visual information, appears to have persisted although the somatosensory input was enhanced. It is thus legitimate to assume that, in order to modify the strategy adopted by these patients, prolonged wearing of somatosensory orthoses would be necessary. The long-term use of somatosensory orthoses would both stimulate and preserve somatosensory receptors and thus develop and consolidate the neural network, supporting a more balanced sensory-motor strategy. Lastly, unlike previous studies which found no effect for PI and CG, our study suggested their efficacy on postural stability in hEDS patients (Hijmans et al., 2009; Dankerl et al., 2016). Indeed, in healthy controls, it is possible that the improved somatosensory input provided by CG actuate more information than needed to control their posture. Hence, the wearing of CG may induce noise in the somatosensory input in healthy subjects, whereas it helps adjust the somatosensory threshold in hEDS patients (Hijmans et al., 2009). Likewise, PI did not induce any effect on postural stability in healthy subjects, probably because no proprioceptive enhancement was required (Dankerl et al., 2016).

### Study Limitations

This pilot study presents a number of limitations. First, the study was conducted on a small sample. Second, the methodology used to investigate SVV could be improved in several respects: (i) the number of trials performed (Piscicelli et al., 2015: a minimum of six trials are required); (ii) the subject’s head should be fixed to their support to prevent any speculative movements; (iii) the head could be placed in the same alignment as the body; and (iv) the lying position could also be performed on the left side.

### Conclusions

Collectively, the functional explorations performed on hEDS patients, using posturography and SVV, suggest an imbalance in the integration of sensory inputs. The results tended to show that somatosensory impairment modifies both verticality perception (Aubert effect) and postural instability. More specifically, results from postural assessment suggest a re-weighting of multisensory integration in favor of visual input. This compensatory strategy, adopted by the patients in order to maintain their balance, may diminish their adaptability, which could, at least in part, account for their postural instability. In contrast, our findings suggest an enhancement of somatosensory feedback induced by the orthoses, thus facilitating postural control, which in turn tends to become more stable. Lastly, this is the first investigation assessing the effect of somatosensory orthoses in hEDS patients, providing new perspectives for improving medical care. However, the observations in this pilot study need to be confirmed by further investigations with a larger number of subjects. Yet, they strongly suggest that postural and SVV assessments are potentially useful tools for the diagnosis and monitoring of this pathology.

## Author Contributions

LMD and EGD designed the study. LMD, EGD and PL carried out the experiment. EGD, LMD and CC analyzed the data. EGD and LMD conceived the figures. EGD, LMD, SB, BB and PD interpreted the results and drafted the manuscript. BB, AS and EV screened potential participants to determine their eligibility for the study. All authors revised the manuscript and approved its final version.

## Conflict of Interest Statement

The authors declare that the research was conducted in the absence of any commercial or financial relationships that could be construed as a potential conflict of interest.
